# Alternative 3′ Pre-mRNA Processing in Saccharomyces cerevisiae Is Modulated by Nab4/Hrp1 In Vivo

**DOI:** 10.1371/journal.pbio.0050006

**Published:** 2006-12-26

**Authors:** Karen S. Kim Guisbert, Hao Li, Christine Guthrie

**Affiliations:** Department of Biochemistry and Biophysics, University of California San Francisco, San Francisco, California, United States of America; University of Oxford, United Kingdom

## Abstract

The Saccharomyces cerevisiae RNA-binding protein Nab4/Hrp1 is a component of the cleavage factor complex required for 3′ pre-mRNA processing. Although the precise role of Nab4/Hrp1 remains unclear, it has been implicated in correct positioning of the cleavage site in vitro. Here, we show that mutation or overexpression of *NAB4/HRP1* alters polyA cleavage site selection in vivo. Using bioinformatic analysis, we identified four related motifs that are statistically enriched in Nab4-associated transcripts; each motif is similar to the known binding site for Nab4/Hrp1. Site-directed mutations in predicted Nab4/Hrp1 binding elements result in decreased use of adjacent cleavage sites. Additionally, we show that the *nab4-7* mutant displays a striking resistance to toxicity from excess copper. We identify a novel target of alternative 3′ pre-mRNA processing, *CTR2,* and demonstrate that *CTR2* is required for the copper resistance phenotype in the *nab4-7* strain. We propose that alternative 3′ pre-mRNA processing is mediated by a Nab4-based mechanism and that these alternative processing events could help control gene expression as part of a physiological response in *S. cerevisiae.*

## Abstract 2

The authors show that alternative 3′ pre-mRNA processing helps control gene expression as part of a physiological response in*S. cerevisiae.*

## Author Summary

A fundamental step in gene expression is the generation of the terminal edge (3′ end) of the mRNA transcript by appropriate cleavage of the longer pre-mRNA. In general, the processing site that emerges first is used, but there are interesting examples where alternative sites are used. Because the choice of alternative sites can add or delete sequences which can affect transcript stability, localization, or translation, it is important to understand how this process is regulated. We have addressed this question in the genetically tractable yeast *Saccharomyces cerevisiae.* Nab4 is a sequence-specific RNA-binding protein involved in 3′ processing. We showed that cells that make too little or too much Nab4 exhibit significant changes in the ratios of alternative 3′ ends. Interestingly, we found that a *nab4* mutant is able to grow on concentrations of copper that are toxic to normal cells. We identified a gene required for this capability, *CTR2,* and showed it has alternative 3′ ends sensitive to the presence of Nab4. We predict that by influencing levels of other alternative 3′ ends, Nab4 is also important for the appropriate biological responses to many other stresses.

##  Introduction

3′ pre-mRNA processing severs the nascent transcript from the elongating polymerase to liberate a new polyadenylated mRNP particle [1,2]. Since the placement of the cleavage site can include or remove RNA sequences that influence transcript stability, transcript localization, protein expression, or protein localization, cleavage site choice can have profound influences on gene expression. In higher eukaryotes, regulation of gene expression via alternative 3′ pre-mRNA processing plays essential roles in development and tissue-specific functions [3–7]. In fact, approximately 50% of human genes are suspected to have alternative polyadenylation sites based on bioinformatics analysis [8,9].

In *Saccharomyces cerevisiae,* the in vivo mechanism of cleavage site selection and the physiological consequences of alternative cleavage remain largely unknown. Only a few cases of multiple polyadenylation sites have been confirmed by having their 3′ ends mapped [10–12]. Interestingly, the site of polyadenylation for half a dozen of these alternatively cleaved transcripts are sensitive to the growth condition of the cell [10,11]; raising the tantalizing possibility that alternative polyadenylation may be dynamic and regulated.

In both yeast and metazoan systems, the multi-subunit cleavage and polyadenylation machinery is assembled on virtually all RNA polymerase II transcripts. The protein components between yeast and mammalian systems that were once thought to be so divergent are now known to be relatively well-conserved [13,14]. In striking contrast, the sequence elements that recruit the cleavage machinery are phylogenetically diverged.

In *S. cerevisiae,* five sequence elements have been identified that contribute to cleavage site selection: the efficiency element, the positioning element, the near-upstream site, the cleavage site, and the near-downstream site [14–16]. However, no single element is absolutely required and each element can be degenerate, making it difficult to accurately predict the 3′ end for most yeast transcripts. These issues are compounded when considering alternative 3′ pre-mRNA processing signals, which may diverge more significantly than a typical 3′-end processing site.

In contrast, the AAUAAA hexamer found in mammalian sequences has long been thought to be an invariant signal for polyadenylation. Interestingly, recent bioinformatics analysis suggests that the variability of mammalian polyadenylation signals may be more akin to those found in S. cerevisiae [17]. Almost a dozen variants to the AAUAAA hexamer have been suggested to play roles in polyadenylation [18]. It has been suggested that the variability in sequences may be utilized as part of the mechanism of alternative 3′ pre-mRNA processing [9,17].

Two known mechanisms of regulated 3′ pre-mRNA processing in metazoans are based upon controlling key components of the cleavage machinery. The best studied example of regulated alternative processing in mammalian cells involves CstF64 and the transcript for immunoglobulin M [4,5]. In resting cells, low levels of CstF64 allow the production of a long form of the transcript that encodes a transmembrane domain, leaving the protein tethered to the cell. Upon B-cell activation, levels of CstF64 rise, which causes a weaker, upstream cleavage site to be used, eliminating the transmembrane domain and creating a secreted protein. The heterodimer CFI_m_ is another component of the mammalian cleavage machinery recently discovered to influence cleavage site selection [19,20]. In fact, CFI_m_ can influence the cleavage site selection of one of its own subunits [19]. Although the consequences of this potential auto-regulation remain unknown, the data underscore the notion that control of a component of the cleavage machinery can regulate cleavage site selection.

The closest S. cerevisiae ortholog of CFI_m_ may be Nab4/Hrp1 [20]. Nab4 is an essential heterogeneous nuclear ribonucleic acid (hnRNP) protein that can shuttle in and out of the nucleus [21]. In addition, Nab4 has been biochemically isolated as part of the cleavage factor complex [22,23]. Although the involvement of Nab4 in 3′ pre-mRNA processing is undisputed, the precise function of Nab4 during this process remains controversial. It is unclear if Nab4 is involved in the cleavage reaction itself or is only required to correctly position the cleavage site. When Nab4 is excluded from in vitro cleavage reactions, the activation of cryptic cleavage sites dramatically increases, leading to the hypothesis that Nab4 is involved in the discrimination between correct and cryptic sites [24].

In addition to its role in 3′ pre-mRNA processing, Nab4 has been implicated in mRNA export and nonsense-mediated decay [25]. Unlike other members of the cleavage and polyadenylation machinery, Nab4 appears to be retained on the message after 3′ pre-mRNA processing and is escorted with the message out of the nucleus. Once in the cytoplasm, it disengages from the transcript and is recycled back into the nucleus by the import receptor Kap104 [26]. It remains unknown whether the roles of Nab4 in export and decay are downstream consequences of its role in 3′ pre-mRNA processing or if they represent independent functions.

To better understand the mechanism and consequences of alternative 3′ pre-mRNA processing in *S. cerevisiae,* we analyzed the function of Nab4. We show that alternative 3′ pre-mRNA processing is sensitive to the levels of this component of the cleavage complex, similar to the regulated cleavage site selection seen in mammalian cells. In addition, we have uncovered an unexpected role of Nab4 and alternative 3′ pre-mRNA processing in the response to toxic copper concentrations. These data support the hypothesis that not only does alternative cleavage occur, but that the control of alternative cleavage is important for cell physiology.

## Results

### Nab4 Mutants Display Altered Cleavage Site Usage In Vivo

We previously identified transcripts preferentially associated with hnRNPs (heterogeneous nuclear ribonucleic acid proteins) on a genome-wide scale. One of the transcripts that co-immunoprecipitated with Nab4 is SUA7 [27]. SUA7, which encodes the transcription initiation factor TFIIB, is known to have two different cleavage sites in the 3′ UTR [11]. To determine if Nab4 could play a role in alternative 3′ pre-mRNA processing in vivo, we analyzed the SUA7 transcript in a *nab4-7* mutant.

**Figure 1:**
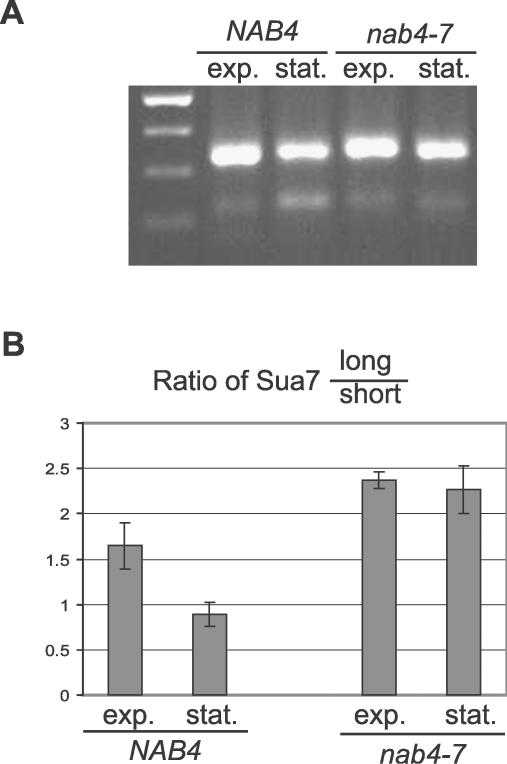
*nab4-7* Displays Aberrant 3′ Cleavage Patterns for the SUA7 Transcript (A) 3′ RACE analysis of total RNA from *NAB4* and *nab4-7* strains in exponential and stationary phase. The SUA7 transcript has two isoforms, long and short. Lane 1 contains a 100-nucleotide molecular weight ladder with the 500-base pair marker located at the top of the gel. Lanes 2 and 3 display 3′RACE analysis of SUA7 from wild-type *NAB4* strains; lanes 4 and 5 from mutant *nab4-7* strains. For lanes 2 and 4, total RNA was harvested from exponential phase, lanes 3 and 5 harvested from stationary phase. (B) Quantitation of the ratio of long versus short form on the SUA7 transcript in *NAB4* and *nab4-*7 strains in both exponential and stationary phase. This quantitation is based upon Northern analysis (unpublished data). Ratio reflects the average of three independent experiments. Error bars reflect the standard error of the mean.

Total RNA samples were collected from *NAB4* and *nab4-7* cells in both log phase and stationary phase. RNA samples were analyzed by 3′ rapid amplification of cDNA ends (RACE) for qualitative analysis (Figure 1A) and by Northerns for quantitative analysis (Figure 1B). Notably, we found that the *nab4-7* strain displays an aberrantly high ratio of the long form to short form for the SUA7 transcript. In our strain background, the ratio of long form to short form is 1.65 in exponentially growing cells. In the presence of the *nab4-7* mutation, the ratio increases to 2.37. This altered ratio is observed at the permissive temperature, where there is no significant growth defect.

Interestingly, in wild-type cells the predominant cleavage site used is sensitive to the growth condition of the cell. The long form predominates during log phase growth and the short form predominates during stationary phase growth [11]. As expected, the control strain displayed the shift from long form to short form during stationary phase growth, reflected in the change in ratio from 1.65 in log to 0.89 in stationary phase (Figure 1). In striking contrast, the *nab4-7* strain showed no appreciable shift in cleavage site usage (a ratio of 2.37 in log phase and 2.27 in stationary phase). Therefore, Nab4 can influence cleavage site usage in vivo for a transcript that is known to have alternative 3′ pre-mRNA processing sites, and a mutant version of Nab4 is defective in a known shift in cleavage site usage.

### Levels of Nab4/Hrp1 Influence Cleavage Site Selection of the SUA7 Transcript

**Figure 2:**
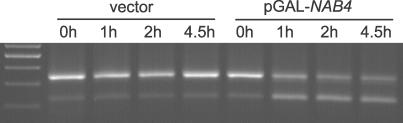
Nab4 Overexpression Affects SUA7 Cleavage Site Selection Shown is the 3′ RACE analysis of the SUA7 transcript during a time course of Nab4 overexpression. Nab4 overexpression was driven by a galactose-inducible promoter. This strain was compared to a vector-only control, induced in parallel. 100-nucleotide molecular weight ladder is shown in the left-most lane with the 600-base pair marker at the top of the gel. Samples were taken before induction and at 1, 2.5, and 4 h post induction.

We next tested if increased expression of an otherwise wild-type version of Nab4 could influence cleavage site selection. Total RNA was collected from strains containing a plasmid with Nab4 under the control of a galactose-inducible promoter*.* Overexpression in the inducible strain was observed by Western blotting; the control strain showed no change in Nab4 levels over the course of the induction (unpublished data). Using 3′ RACE analysis, we found that as Nab4 is overproduced, the relative ratio of long SUA7 transcript to short transcript sharply decreases (Figure 2). Therefore, as excess Nab4 is produced, cleavage at the upstream site to produce a shorter transcript is favored. The shift in cleavage site usage is not due to the change in carbon source, as the addition of galactose to an otherwise identical strain without overexpressed Nab4 shows no such shift in SUA7 cleavage. Therefore, the cellular concentration of wild-type Nab4 can influence cleavage site selection in vivo.

### Potential Nab4-Binding Elements Are Common in 3′ UTR Sequences

In the simplest scenario, Nab4 would exert its influence on cleavage site selection through a direct protein-RNA interaction with the substrate transcript. To identify potential Nab4-binding sites, we took an unbiased bioinformatics approach using the word-finding algorithm Regulatory Element Detection Using Correlation with Expression (REDUCE) [28]. This analysis was similar to previous work in which we identified transcripts that were preferentially associated with Nab4 using an IP-microarray approach [27]. However, in the current analysis, the Ty and Y′ elements that are associated with multiple RNA-binding proteins were eliminated from the dataset prior to REDUCE analysis. We used the algorithm on this filtered dataset to identify short words, up to seven letters long that were overrepresented in transcripts associated with Nab4. The genome was searched from 600 bases upstream to 300 bases downstream of every gene contained in our microarray dataset. This region has been estimated to include the 3′ UTR for 98% of the genome [16]. Motifs that were identified in three independent microarray-REDUCE analyses are shown in Table 1 with the lowest *p*-value calculated for the given motif.

**Table 1:**
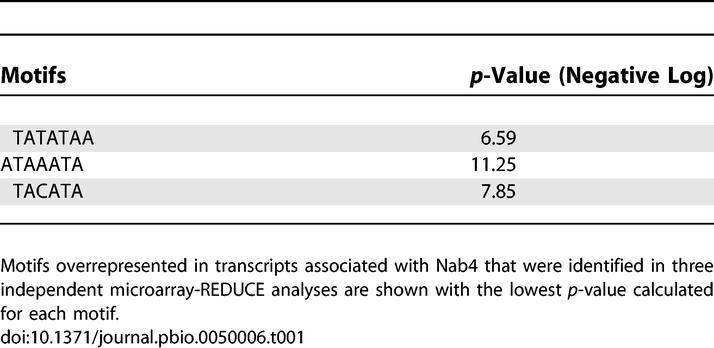
Motifs Associated with Nab4/Hrp1

We identified the word “TATATAA” that is nearly identical to the known Nab4-binding motif of “UAUAUA.” In addition to the TA-rich motif, two other related motifs were identified that contain single nucleotide deviations from the core UA-repeat motif of the known Nab4-binding site, “ATAAATA” and “TACATA.”

**Figure 3:**
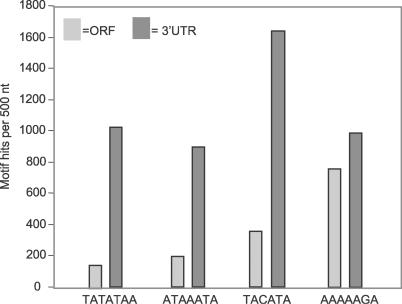
Distribution of Nab4 Motifs in the Genome Is Biased Toward the 3′ UTR Graph displaying the occurrence of Nab4 motifs throughout the genome per 500 nucleotides. Occurrences in ORF sequences are in light gray; 3′ UTR hits are in dark gray. Another sequence (AAAAAAG) associated with Nab2 was included for comparison. nt, nucleotide.

The core UA-repeat motif occurs more frequently in UTR sequences than in open reading frame (ORF) sequences. The deviant motifs display a similar bias as the more canonical motifs in their occurrence in UTR sequences, indicating that the deviant motifs could also be used as 3′ pre-mRNA processing elements (Figure 3). The bias to noncoding regions is not likely due to the AT bias of the S. cerevisiae genome, as a similar motif identified for the hnRNP protein Nab2, “AAAAAAG,” showed no such 3′ bias.

### Nab4 Motifs Promote Cleavage Site Usage

**Figure 4:**
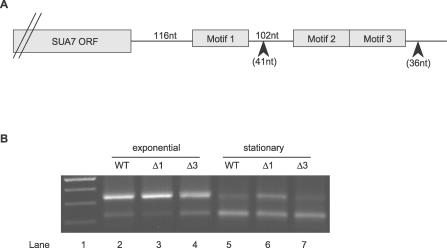
Removal of Nab4 Motifs Alters Cleavage Site Selection (A) Schematic of the layout of Nab4 motifs in the SUA7 3′ UTR. Motifs are numbered from their distance from the stop codon. The median position of the cleavage sites as mapped by Hoopes et al. is noted by an arrowhead. The distance from the adjacent upstream motif is noted in parentheses. (B) 3′ RACE analysis of the SUA7 transcript with motif mutations from wild-type. The Nab4 motifs were removed from SUA7 and replaced with a heterologous sequence. 3′ RACE was performed on strains expressing only the mutant version of SUA7. Both log phase (lanes 2–4) and stationary phase (lanes 5–7) were analyzed. Δ1, motif 1 mutated; Δ3, motif 3 mutated. nt, nucleotide; WT, wild-type.

Given that our analysis implicated Nab4 in cleavage site selection in vivo, we wanted to determine if our newly found Nab4 motifs contributed to cleavage site selection as well. To do so, we created mutations in the potential Nab4-binding sites from the SUA7 3′ UTR. The SUA7 transcript contains three potential Nab4-binding sites that are upstream of the known cleavage sites. The first motif, “AAAAAT,” is located 116 nucleotides from the stop codon and is almost identical to a Nab4 motif identified in our bioinformatics analysis, “ATAAATA.” The second motif “TACATA” and the third motif “TATATATATA” lie directly adjacent to each other, 224 nucleotides from the stop codon. The second motif is identical to a motif identified above and the third motif is an extended form of the canonical efficiency element (Figure 4A). Six nucleotides of each motif were replaced with an unrelated sequence. By replacing instead of removing the motif, the spacing in the primary sequence was preserved between other elements in the 3′ UTR. Due to technical reasons, we were unable to obtain a deletion of the second motif.

Total RNA was collected from strains containing a plasmid encoding the only copy of the *SUA7* gene, and 3′ RACE analysis was performed. As shown in Figure 4, the removal of either the first motif or the third motif resulted in a decrease in cleavage at the adjacent downstream cleavage sites (Figure 4). Mutation of motif 1, the first motif downstream of the stop codon, resulted in a relative decrease in the amount of short form (Figure 4B, lane 3). Likewise, mutation in motif 3, the farthest from the stop codon, resulted in a relative decrease in the amount of long form (Figure 4B, lane 4). The relative shifts in cleavage site usage due to mutations in the Nab4 motifs are apparent in both log and stationary phase (Figure 4B). Since cleavage at adjacent sites are reduced, but not eliminated, the Nab4 motifs cannot be absolutely required to elicit cleavage. Considering that multiple sequence and protein elements work in concert to determine cleavage, the lack of a strict requirement of the element to regulate cleavage site usage is perhaps not surprising. Nonetheless, these data support a role for the influence of Nab4 on cleavage site selection in vivo.

Although 3′ pre-mRNA processing reactions uncovered a role for Nab4 in establishing correct cleavage in vitro, two non-mutually exclusive mechanisms have been proposed: Nab4 could actively promote correct cleavage or it could inhibit incorrect cleavage. Our in vivo results suggest that the Nab4 motifs promote cleavage events.

### Alternative Cleavage via Nab4 May Play a Physiological Role during Copper Stress

**Figure 5:**
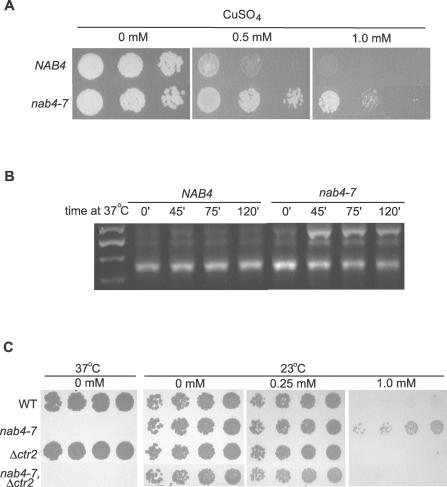
Nab4 and Alternative 3′ Pre-mRNA Processing Plays a Role during Copper Stress (A) Serial dilutions of *NAB4* and *nab4-7* strains on plates containing 0, 0.5, and 1 mM CuSO_4_. Cells were grown at room temperature which is the permissive temperature for the *nab4-7* strain. (B) 3′ RACE of the CTR2 transcript during a shift to 37 °C of *NAB4* and *nab4-7* strains. Samples were taken at 0, 45, 75, and 120 min after shifting both strains to 37 °C, the non-permissive temperature of the *nab4-7* strain. The RACE analysis displays three isoforms which correlate with the presence of three Nab4 motifs in the CTR2 3′ UTR. 100-base pair molecular weight markers are shown in lane 1 with the 300-base pair ladder visible at the bottom of the gel. (C) Serial dilutions of the wild-type *NAB4,* the single mutant *nab4-7,* the single mutant Δ*ctr2,* and the double mutant *nab4-7 Δctr2* strains. The plating conditions are 23 °C, 37 °C, 1 M CuSO_4_, and 0.25 M CuSO_4_. WT, wild-type.

If alternative cleavage site selection is important for a physiological response, then *nab4* mutants that alter cleavage site usage may be defective for that particular response. Interestingly, Nab4-associated transcripts containing the “TACATA” motif are enriched in the functional category of transition metal ion transport, as defined by the Gene Ontology term finder available on the *Saccharomyces* Genome Database (http://www.yeastgenome.org) (unpublished data). To determine if Nab4 plays a role in the expression of genes in this category, we tested if the *nab4-7* mutant displays a growth phenotype in the presence of excess copper. The response to copper stress is an ideal assay as cells are sensitive to both the absence and the excess of copper. Serial dilutions of *NAB4* and *nab4-7* mutant strains were grown on a series of CuSO_4_ -containing plates at room temperature. Surprisingly, the *nab4-7* mutant displays a striking resistance to high levels of copper (Figure 5A). Even in the presence of 1 M CuSO_4_, the *nab4-7* mutant displays robust growth in comparison to its wild-type counterpart (Figure 5A, right panel). The *nab4-7* mutant strain shows no apparent growth defect in normal growth conditions (trace copper) (Figure 5A, left panel). Therefore, we have identified a previously unknown role for Nab4 in copper homeostasis.

With a striking growth phenotype in hand, we hypothesized that the resistance of the *nab4-7* strain to excess copper may be due to a defect in alternative 3′ pre-mRNA processing. To identify alternatively processed transcripts that may play a role in the copper phenotype, we used microarrays to visualize genome-wide changes in transcript abundance in the *nab4-7* strain. We reasoned that an alternate cleavage event could change the stability of the given transcript. Total RNA from *NAB4* and *nab4-7* strains at high-temperature and stationary phase growth were analyzed by standard yeast ORF microarray analysis (unpublished data). Hundreds of genes displayed significant changes in the *nab4-7* background. Notably, the CTR2 transcript was severely affected, showing an increase in transcript levels of more than 8-fold. CTR2 encodes for a vacuolar membrane protein that helps to maintain proper intracellular levels of copper by controlling the flux of copper between the vacuole and the cytosol [29,30].

Given the surprising robustness of the *nab4-7* strain to copper stress and given that the major transcript affected by Nab4 depletion was a gene encoding a copper transporter; we tested if the CTR2 transcript could be an alternatively processed substrate of Nab4. The *CTR2* gene contains three Nab4 motifs at 188, 426, and 581 nucleotides downstream of the stop codon. The first two motifs contain the TATATA motif and the farthest motif contains the TACATA motif. 3′ RACE analysis confirms the presence of three isoforms of the CTR2 transcript (Figure 5B). All three isoforms were confirmed to be CTR2 products using two additional PCR primers in independent reactions (unpublished data).

We found that as functional Nab4 is depleted by a temperature shift of a strain containing the *nab4-7* allele, the ratio between the forms changes, dramatically increasing the proportion of the longest form (Figure 5B). These results add CTR2 to the growing list of transcripts with multiple 3′ pre-mRNA cleavage sites. Moreover, these results demonstrate that the 3′ pre-mRNA processing of the CTR2 transcript is sensitive to the presence of functional Nab4.

Since CTR2 was the most dramatically affected transcript of the copper regulon affected by the *nab4-7* mutation, we hypothesized that it may be involved in the strong resistance of the *nab4-7* strain to excess copper. To test this hypothesis, we created a strain containing either the *NAB4* or the *nab4-7* allele and a deletion of the *CTR2* gene. Serial dilutions of these strains were grown on a series of copper plates (Figure 5C). In the wild-type strain background, deletion of *ctr2* conferred no resistance or sensitivity to excess copper (Figure 5C, second panel from left). Deletion of *ctr2* has no effect on the temperature-sensitive phenotype of the *nab4-7* mutant (Figure 5C, left panel). However, the *ctr2Δ* completely suppresses the *nab4-7* resistance to copper stress Figure 5C, right two panels). These data show that *ctr2Δ* is epistatic to *nab4-7* with respect to copper stress. Therefore, CTR2 and possibly its alternative 3′ pre-mRNA processing play a central role in the copper resistance phenotype of the *nab4-7* strain.

## Discussion

We have analyzed the role of Nab4 in alternative 3′ pre-mRNA processing. We have shown that both mutation and overexpression of Nab4 can influence cleavage site selection in vivo*.* We have shown that the *nab4-*7 strain shows a striking resistance to excess copper. Moreover, we have identified that the CTR2 transcript has alternative 3′ ends and is required for the resistance of the *nab4-7* strain to excess copper. We propose that alterations in the level or activity of Nab4, a component of the cleavage machinery, can be used to achieve diverse physiological responses.

### Implications for Gene Regulation

Alternative cleavage site selection may be more common and more important than is currently appreciated. Based on our microarray and bioinformatic analysis, we have identified a previously unknown role for Nab4 in copper homeostasis. In our previous work, we identified other functional categories of transcripts that are preferentially associated with Nab4 [27]. We anticipate that transcripts in these other functional categories will also display physiological consequences if their 3′ pre-mRNA processing is misregulated. In addition, estimates of alternative cleavage events were taken from EST data from a single phase of growth. Since alternative cleavage events can be sensitive to the growth condition of the cell, these estimates are likely to be low [10,11]. Between the three motifs identified here and the previously known canonical Nab4-binding element, approximately 59% of transcripts in the yeast genome contain more than one Nab4 motif in the region encompassing the ORF and 500 nucleotides downstream of the stop codon (unpublished data). It would not be surprising if the number of actual alternative cleavage events rivals the 54% currently predicted for the mammalian transcriptome [9].

Although we have concentrated our analysis on Nab4, we suspect that other players also contribute to regulated 3′ pre-mRNA processing. The work of Minvielle-Sebastia that originally motivated this work also implicated CF1a, another member of the cleavage complex, in establishing proper cleavage [24]. Additionally, it has been suggested that a third member of the cleavage complex, Rna14, affects the 3′-end processing of its own transcript [12]. It will be interesting to determine if other members of the cleavage complex can also influence cleavage site choice in vivo similar to Nab4.

### Alternative 3′ Pre-mRNA Processing and the Copper Response

We have identified a correlation between alternative processing, Nab4, and the copper response. We have shown that a *nab4-7* strain displays a striking resistance to excess copper and that this resistance requires the presence of the *CTR2* gene. We demonstrated that the CTR2 transcript has multiple 3′ ends and that the cleavage site used is sensitive to the presence of functional Nab4. Interestingly, this exceptionally long 3′ UTR, almost 600 nucleotides, places the CTR2 transcript in the top 2% of predicted yeast 3′ UTR lengths. The average yeast transcript is estimated to have a 3′ UTR of less than 100 nucleotides [16]. Moreover, the quantity of CTR2 transcript in the cell rises dramatically as the *nab4-7* mutant strain is shifted to the restrictive temperature. Exactly how the alternative processing affects the function of CTR2 and how this allows the *nab4-7* mutant cells to resist excess copper remains unknown. Whether the alternative 3′ pre-mRNA processing of CTR2 is directly responsible for the copper phenotype in unknown; however, the correlation between Nab4, CTR2, and alternative 3′ pre-mRNA processing is too enticing to ignore.

Interestingly, the 3′ UTR has recently been discovered to play an important role in another metal-stress responsive pathway. During iron-stress conditions in *S. cerevisiae,* the cell relies on mRNA surveillance mechanisms to respond to low levels of iron. In iron-replete conditions, specific mRNAs are targeted for degradation via sequence elements in the 3′ UTR of target transcripts [31]. Additionally, the absence of RNA surveillance pathways leads to sensitivity to high iron conditions in S. cerevisiae [32]. It remains to be seen if alternative 3′ pre-mRNA processing of CTR2 plays into a similar mRNA surveillance pathway, as seen in iron stress. Nonetheless, the importance of the 3′ UTR in different stress conditions emphasizes the potential for alternative 3′ pre-mRNA processing to effect cell physiology.

Our study of alternative 3′ pre-mRNA processing illustrates the utility of studying this process in a genetically tractable organism. Given our unexpected discovery of a role for Nab4 in copper toxicity, we suspect that other growth and stress conditions will reveal even more regulated, alternative processing events. Moreover, identifying the mechanism and consequences of regulated 3′ pre-mRNA cleavage has proven time-consuming in metazoan cells. In *S. cerevisiae,* this type of analysis should be more facile than in metazoan cells.

### Conservation of Mechanism

Just as the machinery of 3′ pre-mRNA processing between yeast and mammals is more closely related than first expected, we hypothesize that the regulation of alternative processing also remains conserved. In the simplest model of alternative 3′ pre-mRNA processing, Nab4 associates with its binding site and promotes cleavage at an adjacent site. Changing the association of Nab4 with a given site would then change the cleavage site to be used.

Known instances of alternative 3′ pre-mRNA processing in mammalian cells, such as for CstF64 and the transcript for immunoglobulin M, are characterized by at least three defining features: first, multiple sequence elements differentially recruit the cleavage complex; second, an RNA-binding protein recognizes a specific sequence element; and lastly, the activity or concentration of the RNA-binding protein is regulated. We have shown that Nab4 fulfills each of these requirements.

Similar to CstF64, Nab4 is a component of the processing machinery. Just as control of the levels of CstF64 is a fundamental component to regulating cleavage site selection in B cells, we have found that the levels of Nab4 can likewise affect cleavage site selection in vivo. Moreover, the association of CstF64 with its binding element leads to activation of a nearby cleavage site. Similarly, we find that deletion of predicted Nab4-binding motifs reduces cleavage efficiency at adjacent sites. These data are not consistent with an alternate model proposed for Nab4 function which suggests that Nab4 association blocks inappropriate cleavage sites [24]. Therefore, just as the protein and sequence elements have been found to be conserved from yeast to mammals, we propose that the regulation of alternative cleavage site selection is similarly conserved.

### Biochemistry of Nab4 Association

A key feature of regulated 3′ pre-mRNA processing in mammalian cells is the ability of CstF64 to discriminate between strong and weak polyadenylation signals. If the mechanism of regulated cleavage is indeed conserved, then we expect Nab4 to also show such a bias between sites. In the simplest case, such a discrimination of the protein would be based on its binding affinity to the efficiency element. We identified three motifs that are enriched in messages preferentially associated with Nab4, one that contains the canonical efficiency element and two that are single nucleotide deviations from the canonical. For the SUA7 transcript, the deviant motif lies upstream of the canonical motif and the upstream site is the less preferred site during log phase growth. We predict that the deviant motifs will display a lower affinity for Nab4 binding than the canonical motifs. Additionally, we have identified just three motifs via our microarray and bioinformatics analysis. Bioinformatics analysis has shown that other single-nucleotide deviations away from the core UA repeat motif are also statistically enriched downstream of ORF sequences [15,16,33]. While the affinity of Nab4 for the canonical efficiency element has been estimated, the specificity of Nab4 remains uncharacterized. It would be interesting to determine if the affinity of Nab4 for these different sequence elements correlates with the ability to undergo alternative 3′ pre-mRNA processing.

### Regulation of Nab4

Based on our findings that the overexpression of Nab4 alters cleavage site selection, we predict that the cell should exert some regulation over Nab4 itself. Moreover, if Nab4 is regulated, then cleavage site selection may also be regulated. Nab4 is already known to be subjected to both methylation and phosphorylation [34,35]. Both modifications have the potential to alter Nab4 function. Another indication of Nab4 regulation is that both too little (*NAB4* is essential) and too much Nab4 lead to cell mortality (unpublished data; M. Swanson, personal communication). In fact, several lines of evidence imply that the levels of Nab4 are continuously adjusted based upon growth conditions. First, NAB4 transcript levels are sensitive to several growth conditions as shown by microarray analysis. Interestingly, NAB4 transcript levels decrease during stationary phase [36], increase during the early phase of sporulation [37], and fluctuate during metabolic cycling in nutrient-limited conditions [38]. In addition to regulating the total concentration of Nab4, the local concentration of Nab4 could be affected. Since cleavage site selection is presumably a nuclear event, changes in the nuclear concentration of Nab4 should affect cleavage site choice as well. Interestingly, certain stress conditions, such as hypo-osmotic stress, can affect the localization of Nab4 to move from mainly nucleoplasmic to largely cytoplasmic [39]. We predict that conditions which change the total or local Nab4 protein levels or the activity of Nab4 will correlate with changes to cleavage site choice in transcripts important in these conditions.

### Concluding Remarks

In this paper, we address two critical issues concerning alternative 3′ pre-mRNA processing: how is alternative cleavage site choice mediated in vivo and what are the physiological consequences of alternative cleavage? We propose a mechanism of regulation via titration of a component of the cleavage machinery that is similar to the known mechanism utilized in mammalian cells. More importantly, this work describes a relationship between alternative cleavage and a physiological response in *S. cerevisiae.* The unexpected discovery of a robust growth phenotype of the *nab4-7* strain to excess copper demonstrates the utility of our approach of identifying preferentially associated binding partners of RNA-binding proteins and using bioinformatics to uncover clues as to the function of the protein. We predict that copper stress is the first of many physiological responses that depend, in part, upon alternative cleavage.

## Materials and Methods

### Strain preparation.

Yeast manipulations were executed according to Guthrie and Fink [41]. The *nab4* mutant strains were generously provided by the Swanson Laboratory and were first described by Minvielle-Sebastia et al.[24]. Each strain has the endogenous *NAB4* gene deletion covered by CEN plasmid carrying either a wild-type or mutant version of *NAB4.* The Swanson Laboratory also generously provided the Nab4 overexpression strain. This strain is a diploid, heterozygous at the *NAB4* locus (one wild-type and one deletion), containing a plasmid with *NAB4* under the control of the Gal4, galactose-inducible promoter. The Δ*ctr2* and *nab4-7,*Δ*ctr2* double mutant strain were constructed in the same strain background as above according to the method in Longtine et al. [40].

### Northerns.

Total RNA was isolated using four organic extractions: once in hot, acidic phenol-choloform; twice in cold, acidic phenol-chloroform; and a final extraction in chloroform-isoamyl alcohol. The RNA was then precipitated with sodium acetate and ethanol and resuspended in water. Equal amounts of RNA by OD were diluted into loading buffer and loaded onto an agarose gel between 1.5%–2% agarose in TBE buffer. RNA was transferred onto a membrane using capillary action for at least 6 h or overnight. Both the RNA on the gel and on the membrane was visualized to ensure successful transfer. The RNA was immobilized on the membrane with UV-crosslinking. Radioactive probes for the Northern analysis were ^32^P end-labeled oligos. Rapid-Hybe buffer (Amersham, http://www.amersham.com) was used during the hybridization. Quantitation was performed using a Storm Phosphorimager.

### 3′ RACE analysis.

Total RNA was isolated using the same procedure described from Northern analysis. 20–25 μg of total RNA was reverse transcribed using Superscript III (Invitrogen, http://www.invitrogen.com) and an anchored dT primer. The cDNA was diluted 10-fold and then PCR-amplified using one primer specific to the ORF and one primer specific to the dT oligo. The primer for the SUA7 analysis was “ataacttaccgggcgttg” and the primer for CTR2 analysis was “tggggcaatatggggtaattaca.” The 3′ RACE reactions were separated on 1.5% agarose gels.

### Copper resistance assay.

Strains were grown to log phase, then diluted to the same density by OD. 3- to 4-fold serial dilutions were prepared and plated onto a series of plates containing various concentrations of CuSO_4_ or rich media (YPD). Except where noted, strains were grown at room temperature for 3–5 d.

## Abbreviations

ORF: open reading frame
RACE: rapid amplification of cDNA ends
REDUCE: regulatory element detection using correlation with expression
